# Evaluating Older Drivers in Their Everyday Driving Environments

**DOI:** 10.1093/geroni/igaa057.2597

**Published:** 2020-12-16

**Authors:** Isabelle Gelinas, Barbara Mazer, Yu-Ting Chen, Brenda Vrkljan, Shawn Marshall, Judith Charlton, Sjaanie Koppel

**Affiliations:** 1 McGill University, Montreal, Quebec, Canada; 2 McGill University School of Physical and Occupational Therapy, Montreal, Quebec, Canada; 3 McMaster University, Hamilton, Ontario, Canada; 4 University of Ottawa, Ottawa, Ontario, Canada; 5 Monash University Accident Research Centre, Clayton, Victoria, Australia

## Abstract

Developing tools that accurately detect at-risk driving behaviors is a public-health priority. There is a need for a measure that accurately assesses older drivers’ level of competence on familiar roadways. The objective of this presentation is to describe the development of the procedures and scoring of a new approach, the Electronic Driving Observation Schedule (eDOS), to observe everyday driving in the community. The eDOS was used to record and compare the driving environment and performance of older drivers and low-risk younger drivers during their everyday driving. Older (n=160, >74y) and younger (n=60, 35-64y) drivers completed a 20-30-minute drive from their home to destinations of their choice. Older drivers drove on simpler routes with fewer intersections and lane changes. Both groups made few driving errors, which were mostly low-risk. Younger drivers tended to demonstrate poor driving habits (not signaling, speeding, poor lane position) and compliance with road rules. Part of a symposium sponsored by Transportation and Aging Interest Group.

